# Cortico-cancellous osseointegration into additively manufactured titanium implants using a load-bearing femoral ovine model

**DOI:** 10.3389/fbioe.2024.1371693

**Published:** 2024-06-24

**Authors:** Adam Feldman, Michel Assad, Mark B. Davies, Jitendra Mangwani, Enrique Alabort, Mac Tuncer

**Affiliations:** ^1^ Alloyed Ltd., Oxford, United Kingdom; ^2^ AccelLAB Inc., Boisbriand, QC, Canada; ^3^ Northern General Hospital, Sheffield, United Kingdom; ^4^ Leicester Royal Infirmary, Leicester, United Kingdom

**Keywords:** additive manufacturing, orthopedic, implant, lattice, ovine, osseointegration, Ti-6Al-4V, mesh

## Abstract

**Introduction:** Titanium-based implants can be used to fill voids in bone reconstruction surgery. Through additive manufacturing (AM), it is possible to produce titanium implants with osteoconductive properties such as high porosity and low stiffness. AM facilitates a level of design flexibility and personalization that is not feasible with traditional techniques.

**Methods:** In this study, osseointegration into titanium alloy (Ti-6Al-4V) lattices was investigated for 12 weeks post-implantation using a novel bicortical load-bearing ovine model. The objective was to assess the safety and efficacy of AM-fabricated implants using two lattice structures of contrasting stiffness spanning the full width of the femoral condyle.

**Results:** This was achieved by evaluating implant osseointegration and bone–implant contact properties by histomorphometry, scoring local implant tissue responses via histopathology, and micro-computed tomography reconstruction.

**Discussion:** We found that Ti-6Al-4V implants facilitated widespread and extensive osseointegration, with bone maturation ongoing at the conclusion of the trial period. Following the implantation period, no adverse clinical indications that could be directly ascribed to the presence of the implanted device were identified, as determined by macroscopic and microscopic observation.

## 1 Introduction

Titanium alloys are widely used in biomedical applications because of their corrosion-resistant, bio-inert, and osseointegrative properties. While titanium-based implants have proven extremely successful across many applications, the minimization of implant stiffness remains a challenge for orthopedic indications. The elastic modulus of human cancellous and cortical bone typically varies from 0.01 to 3 GPa and from 3.3 to 21.8 GPa, respectively (Morgan et al., 2018). This stands in contrast to the elastic modulus in the region of 110 GPa for titanium aluminum vanadium alloy (Ti-6Al-4V) (He et al., 2020). A significant discrepancy between implant and tissue stiffness can result in stress shielding due to decreased physiological loading of the bone surrounding an implant (Niinomi and Nakai, 2011). The subsequent remodeling resulting from stress shielding can be detrimental to bone structural integrity, ultimately leading to osteoporosis and aseptic loosening of the implant (Niinomi and Nakai, 2011).

Additive manufacturing (AM) allows for a high degree of morphological flexibility in the design of titanium-based implants. This geometrical freedom opens possibilities for new clinical applications and provides an opportunity to address challenges, such as osseointegration and stress shielding, through topology optimization.

The literature describes the suitability of laser powder bed fusion (LPBF) for implant manufacturing due to the superior corrosion resistance and surface finish of the printed article (Zadeh et al., 2022). Additionally, Pilz et al. described a process for optimizing Young’s modulus in parts manufactured using LPBF through microstructural texturing (Pilz et al., 2022). They recorded a reduction of over 30% in Young’s modulus in a ß-type Ti-42Nb alloy, while retaining material yield strength. This demonstrates potential for LPBF as an orthopedic implant manufacturing process (Pilz et al., 2022).

Titanium-based, custom-made implants can be designed to conform to a patient’s anatomy or intended surgical geometry. Each implant is unique and can be prepared to fill large bone cavities caused by trauma, tumor removal, revision surgery, deformities, and osteonecrosis or for other types of bone reconstruction. The open structure of the implant provides a scaffold into which bone autografts can be packed to facilitate tissue regrowth and fusion.

The sample implants used in this study were made of titanium-6aluminum-4vanadium (Ti-6Al-4V), consisting principally of struts (diameters between 1.1 and 2.0 mm) and curved connector members (connecting to adjacent struts), forming an open-cell, cage-like structure. The structure was designed to minimize mechanical stress raisers and the appearance of small cavities (with a high radius of curvature). In this study, the safety and osseointegrative properties of titanium-based cage-like implants were investigated using a novel bi-cortical, load-bearing ovine model.

## 2 Materials and methods

### 2.1 Experimental design

The primary objective of this pilot study was to investigate the osseointegrative properties of titanium-based cage-like implants. Two implant designs were specified for implantation. The experimental parameter used was variation in strut thickness to investigate its optimization in facilitating bone ingrowth. Strut thickness will also have an impact on the stiffness of the overall device. As such, despite this study not directly investigating the effect of stiffness on osseointegration, two different devices could be considered to represent a high- and a low-stiffness device. Using an ovine model, a sample was implanted in both the right and left femoral metaphyses. The study lasted 12 weeks, throughout which clinical manifestations were monitored and recorded. Following the scheduled termination date, a histopathological examination was performed *via* macroscopic and microscopic analyses of explanted materials to evaluate the local implantation effects and the pathological response. Histomorphometry was also undertaken to quantify the extent of osseointegration into the porous scaffold. Test (TA) and control (CA) article allocation is described in Table 1.

**TABLE 1:** Distribution of test (TA) and control (CA) implants.

Animal number	Left femoral condyle		Right femoral condyle	
	Individual test material allocation	Length of the device (mm)	Individual test material allocation	Length of the device (mm)
D84-01	TA	37	TA	37
D84-02	CA	37	TA	38
D84-03	TA	40	TA	37
D84-04	TA	37	CA	38

### 2.2 Implant description

Cylindrical, custom titanium-based implants (CTBIs) (diameter 11.2 mm and length ranging from 28 to 42 mm; 15 variations) (Meshworks, Oxford, United Kingdom) were first manufactured using laser powder bed fusion (LPBF). The samples were designed using Rhinoceros^®^ (Robert McNeel & Associates) with a Voronoi exterior and body centered cubic interior lattice generated stochastically with a target relative density and node valence of 17.77% and 3.76, respectively. They were manufactured using a Renishaw AM250 (Renishaw, United Kingdom). Laser power for fill and borders was 200 and 150 W, respectively. Layer thickness was maintained at 30 μm, hatch distance at 105 µm, and hatch point distance was at 55 µm. The samples were manufactured using grade 23 Ti-6Al-4V extra-low interstitial powder (Carpenter Technology Corporation). The implants were then heat-treated and blasted to manually remove struts.

**FIGURE 1:**
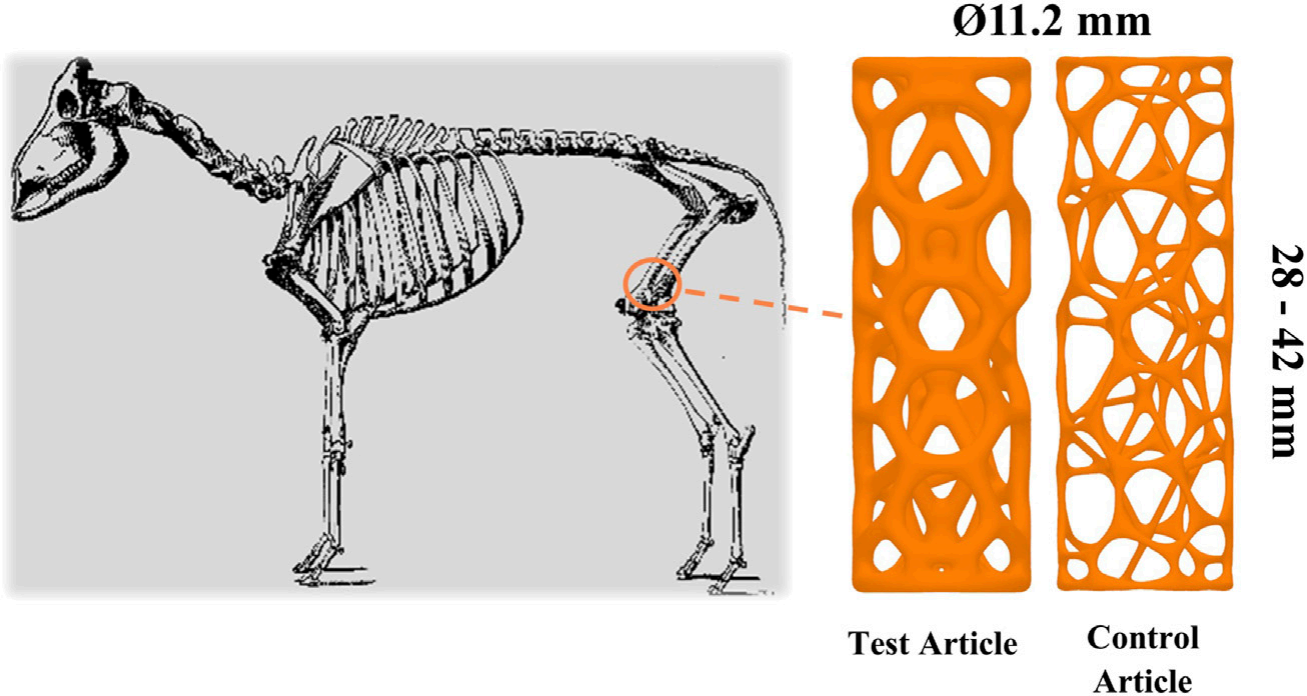
Depiction of implant geometry and the surgical site.

Cylindrical representations of a CTBI were chosen as they facilitated a minimally invasive surgical procedure due to ease of bone preparation and insertion. The variation in length provided intraoperative flexibility and accommodated variation in femoral condyle length, which was critical given that the implant had to occupy a bi-cortical defect. The length and type of the implanted article is outlined in Table 1. The strut thickness of the TA and CA was 1.3 and 0.65 mm, respectively. The implants underwent compression testing prior to implantation with a failure load of >10 and 2.5 kN, respectively, for devices with a strut thickness of 1.3 and 0.65 mm, respectively. This confirmed the implants would not fail under typical loading *in vivo*. The implants were provided cleaned but non-sterile to the test facility. Accordingly, they were subsequently autoclaved prior to implantation, which was performed using an ovine long-bone model. Figure 1 depicts the TA, CA, and intended site of implantation.

### 2.3 Model description

#### 2.3.1 Model selection justification

Four skeletally mature female Dorset sheep (*Ovis aries,* Hybrid Arcott Dorset; 3–5 years old, 56–63 kg; Alpha Ovine, Norwood, ON, Canada) were simultaneously prepared for bilateral hind limb surgery. Despite the contrasting anatomy of quadrupeds and bipeds, sheep are useful models for addressing the biomechanical, biochemical, and histological processes of bone biology of bipeds, given the similarities in scale, weight, joint structure, and bone remodeling processes between the two groups (Potes et al., 2008). Ovine-based studies of similar design are commonly reported in literature. Sheep are a convenient large-animal model for biomedical research owing to their wide availability, ease of handling, low cost, and acceptance by society as a research animal (Potes et al., 2008). The large anatomical scale of this model also allows for accurate visualization of the tissue surrounding the implant using fluoroscopic imaging as well as the use of standard catheterization equipment.

### 2.4 Surgical procedure

The protocol was reviewed and approved by the Testing Facility’s Institutional Animal Care and Use Committee (IACUC). The review ensured compliance with Canadian Council on Animal Care (CCAC) regulations. The testing facility is accredited by the Association for Assessment and Accreditation of Laboratory Animal Care (AAALAC) and the CCAC.

The animal was anesthetized intravenously with a mix of midazolam (0.5 mg/kg), ketamine (5 mg/kg), and glycopyrrolate (0.01 mg/kg). This was calculated by the most recent practical body weight available from a fed animal from the testing facility’s spare colony. The larynx was sprayed with lidocaine, and the animal was intubated with an appropriately sized, cuffed orotracheal tube. When intubation was not possible, induction was completed using propofol (3.5 mg/kg IV, to effect). After tracheal intubation, the sheep was mechanically ventilated with isoflurane in oxygen.

The ipsilateral hind limb was flexed to a position at which the medial condyle could be palpated under the skin. An approximate 6-cm medial parapatellar skin incision was made. After blunt dissection of the subcutaneous tissues, the fascia overlying the vastus medialis muscle was incised distal to the muscle belly, and the vastus medialis was retracted. Blunt dissection was used to expose the periosteum down to the medial condyle of the femur. A periosteum elevator was used to prepare the location of the drilled hole.

A guidewire was then inserted through the femoral condyle under fluoroscopic guidance to ensure accurate alignment prior to drilling. Using the guidewire, the condyle was step-drilled cortex-to-cortex, perpendicular to the shaft of the femur, using increasingly sized reamers (to 11 mm). Bone remnants from the drilling were removed and collected into a cut syringe to approximate the volume of bone fragments obtained. The bone fragments formed a putty-like substance that was pushed into the pores of the devices to be implanted. Once filled with bone fragments, the sample CTBI was inserted into the defect and tapped gently into place with a hammer. The placement of the implant was confirmed through fluoroscopic imaging.

Following TA/CA implantation, the access sites were ligated as necessary, and the incision layers were sutured, as required. After wound closure, a transparent film dressing spray (Opsite; Smith and Nephew, Watford, United Kingdom) was applied to the surgical wound. The same procedures were performed for the contralateral femoral condyle. Following the surgical procedures, the animals were allowed to recover with full weight bearing for 12 weeks after implantation in a climate-controlled room. Health checks were performed daily.

### 2.5 Implant and tissue sample harvesting

Most of the tissue surrounding the distal femur was removed, with care being taken to leave a small amount of soft tissue (∼5–10 mm) overlying the medial portion of the implant. Macroscopic observations undertaken during necropsy procedures or tissue collection were documented. The collected tissues were fixed by immersion in 10% neutral-buffered formalin (NBF). After gross evaluation, the femur was removed and cut with a diamond saw or another appropriate tool, leaving enough of the distal femur to be able to trim the sections relative to the longitudinal axis of the femur. The medial condyle was marked with tissue ink. The distal femur was placed in 10% NBF. The popliteal and inguinal lymph nodes (right and left) were also collected and immersion-fixed in NBF.

#### 2.5.1 Micro-computed tomography (micro-CT)

**FIGURE 2:**
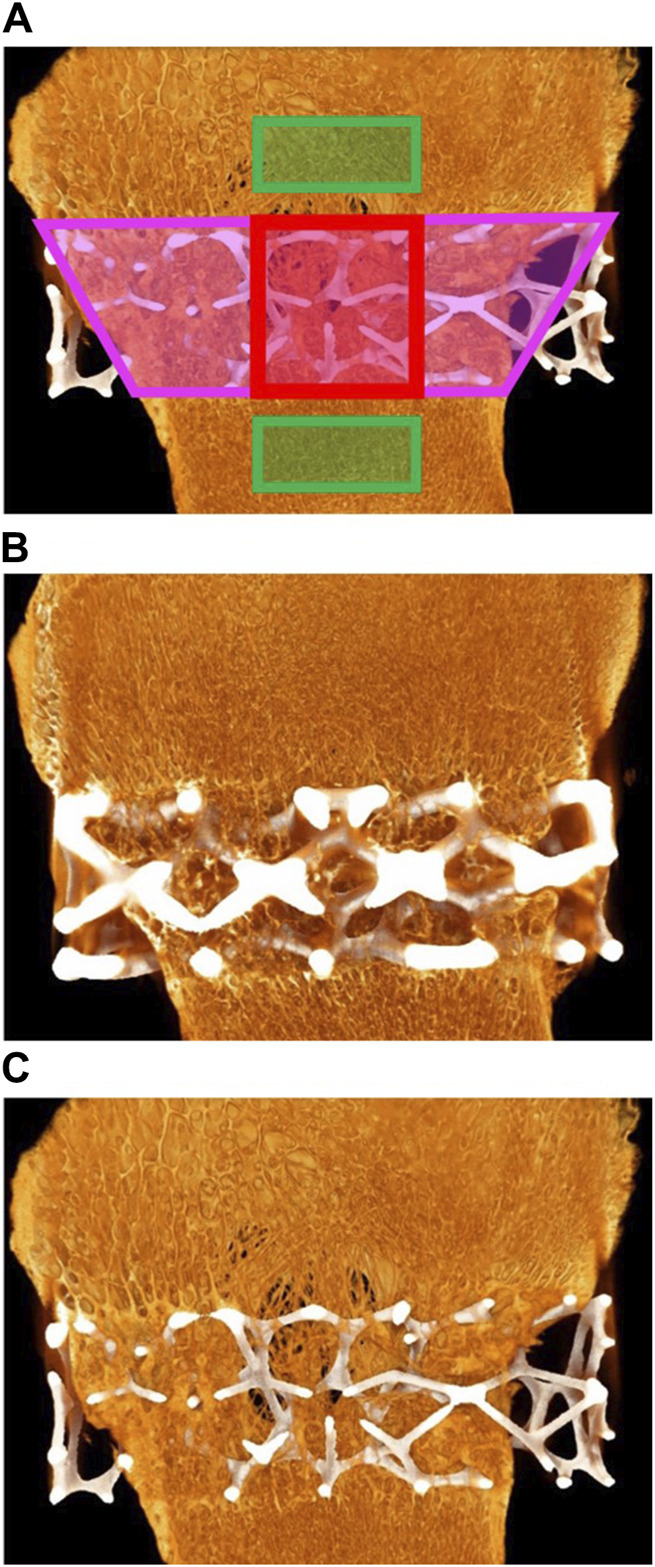
**(A)** Red areas: middle section; pink areas: defect section; green rectangles: outer section. **(B)** Micro-computed tomography (micro-CT) of animal D84-03. Right femoral condyle. Section L-1. Test article. Widespread and extensive bone ingrowth is evident. **(C)** Micro-CT of animal D84-04. Right femoral condyle. Section L-1. Control article with reduced strut size. Widespread and extensive bone ingrowth is evident.

A total of seven micro-CT scans (five TAs and two CAs) of the femoral medial condyles were analyzed. One sample was lost to a hard drive system failure and therefore could not be analyzed (left femoral condyle—D84-01). Micro-CT of the implantation sites was performed using the Nikon XT H 225 micro-CT system at a resolution of 25 µm/vx. Exposure time was set to 1415 ms, and a 1-mm copper filter was used. The samples were scanned at room temperature and then again immersed in 10% NBF. The defect volume of interest (VOI) was defined as the delineation of the implanted cage, the middle VOI was defined as the middle portion of the cage, and the outer VOI was defined as a region outside the cage (i.e., adjacent pre-existing bone) that was virgin to any surgical intervention. The outer section was used as a normal-bone control. Following the identification of the VOIs, the TA/CA volumes were subtracted from the VOIs (when applicable), and the remaining volume was defined as the “Total Volume” (TV) (i.e., the volume within the VOI excluding the TA/CA volume if present). The volume of bone (“Bone Volume”; BV) within the VOI was then determined *via* thresholding. Within each VOI, the BV, TV, average trabecular separation (Tb.Sp), and average trabecular thickness (Tb.Th) were measured, and the BV/TV ratio was calculated. The BV/TV ratio is a measure of the proportion of mineralized bone present in a given area. The Tb.Sp is the major diameter of the cavities formed by the porous microarchitecture of trabecular bone and is an indicator of trabecular health. Tb.Th refers to the diameter of the trabeculae found in a sample of trabecular bone and is an indicator of the extent of trabecular remodeling and overall tissue strength. Figure 2A provides a depiction of the analyzed regions and Figures 2B, C show micro-CT images for the TA and CA, respectively. It should be noted that the presence of the implant within the defect and middle sections may artificially elevate (minimally) the measured BV relative to that of the outer section owing to the artifact produced by the implant. Similarly, owing to differences in size and/or shape between the TA and the CA, the measured BV within the defect and middle sections in the case of the TA may be artificially elevated (minimally) compared with that of the CA. This is as a result of a minor attenuation of X-rays entering the cage, which can lead to an increased apparent volume of bone present.

### 2.6 Sample processing and analysis

#### 2.6.1 Histological processing of condylar implants and soft tissue

**FIGURE 3:**
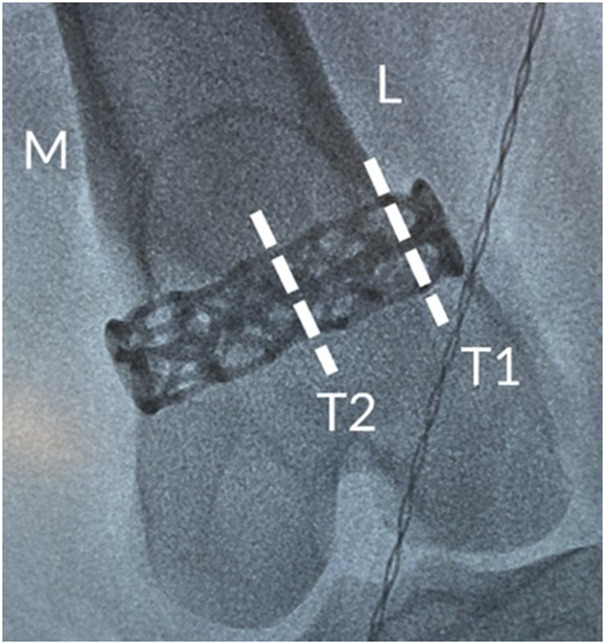
Intraoperative radiograph showing the positioning of the cage-like additively manufactured implant in the distal femur including details of transverse analysis sections.

The explanted samples and surrounding adjacent hard tissue were then embedded in methyl methacrylate, polymerized, and processed for non-decalcified bone histology. The femur was sectioned transversely through the middle portion of the implant along the longitudinal axis of the femur. The implant within the medial portion of the femur was sectioned longitudinally through the center (L1) along the longitudinal axis of the femur. The implant within the lateral portion of the femur was cut into two transverse sections, one at the center of the implant (T2), while the other, targeting the endocortical region, was located at a constant distance from the edge of the bone (T1). These locations are depicted in Figure 3. The distance from the cortex was measured to attempt consistent cuts in all study femurs. The angle of the cut followed, as close as possible, the angle of the lateral femoral cortex in parallel (T1); this may have resulted in an elliptical section of the implant. Two slides were prepared at each level (L1, T1, and T2), and the proximal and distal sides of the slides were noted. One slide was stained with Stevenel’s blue (SB) and the other with hematoxylin and eosin (H&E).

Soft tissues adjacent to the medial portion of the implant were embedded with the implant. In addition, lesions and inguinal lymph node tissues collected during necropsy were embedded in paraffin, sectioned into approximately 5-μm-thick slices, and stained with H&E for histopathological evaluation. All stained sections were examined by a pathologist for semi-quantitative and descriptive histopathology.

#### 2.6.2 Histopathology

H&E− and SB-stained sections were evaluated by the study pathologist to determine the safety and efficacy of the test and control implants (TA/CA). Stevenel’s blue was selected as it allows differentiation between new bone growth and pre-existing tissue (Jackson et al., 2019). SB enables histomorphometric quantification of osteoid tissue, which gives an accurate indication of the maturation level of newly formed osseous tissue (Jackson et al., 2019). Histologic examination using H&E staining included the assessment included the assessment of new bone formation, necrosis/osteolysis, fibrinous exudate, inflammatory cells, and fibrous tissue formation. Additional parameters examined in the undecalcified sections included bone–implant apposition, void spaces, fluid/material, and implant irregularity. Finally, when appropriate, lesions and inguinal lymph nodes were qualitatively graded using the scale shown in Table 2 as a guide.

**TABLE 2:** Histopathology evaluation grading system.

Value	Description
**0**	None
**1**	Minimal: observation was barely perceptible microscopically and was not believed to have clinical significance
**2**	Mild: the pathological feature was visible but involved a minor proportion of the tissue, and the clinical significance was minimal
**3**	Moderate: the pathological feature was clearly visible and involved a significant proportion of the tissue; it was likely to have some clinical manifestations, but these were generally expected to be minor
**4**	Marked: the pathological feature was clearly visible and involved a major proportion of the tissue; clinical manifestations were probable and may have been associated with significant tissue dysfunction or damage

## 3 Results

### 3.1 Clinical observation

During the 12-week study period, none of the animals showed adverse clinical signs that could be directly attributed to the bilateral implantation of a CTBI.

### 3.2 Pathology

#### 3.2.1 Macroscopic observations

Limited necropsies, which included macroscopic examination of the implanted area and surrounding tissue, were performed on all the animals. Dark areas were occasionally observed at both the control and test implant sites. When observed on the femoral condyle, the discoloration showed no microscopic pathological correlate. In the right synovial membrane of one animal, a 5 × 6 mm dark area correlated microscopically to focal, slight erosion with granulation tissue formation. This change was consistent with a healing response following mechanical trauma and was not directly ascribable to the TA.

Another animal showed a red/purple and enlarged left inguinal lymph node. There was no pathological correlate to the macroscopic observation of enlargement and the discoloration corresponded to the presence of blood in the sinuses, which was likely a terminal event.

#### 3.2.2 Histopathology

**FIGURE 4:**
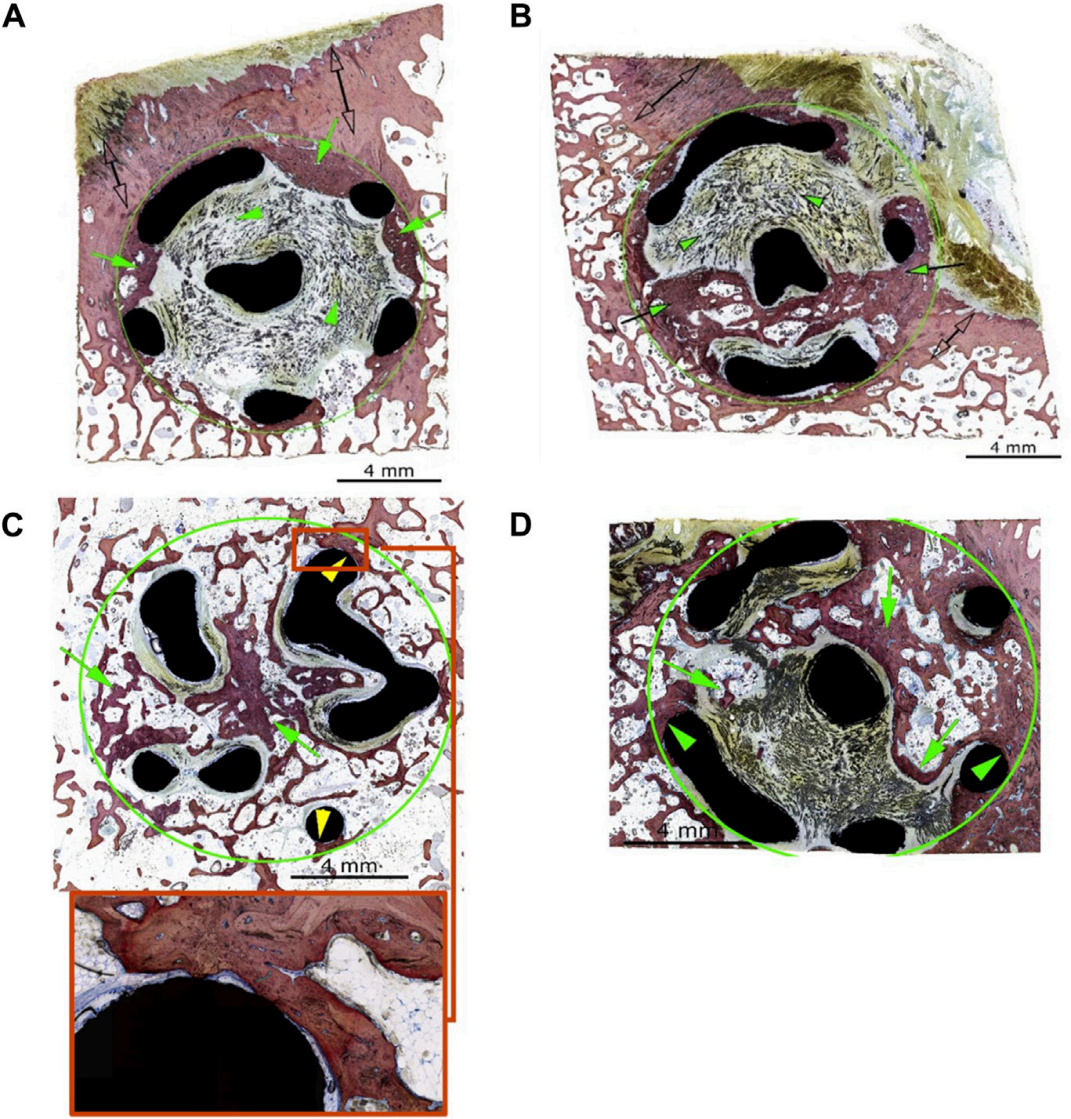
**(A)** Animal D84-04 (SB). Left femoral condyle. Section T-1. Test article. Post-implantation day 83. Green circle: implant site outline; green arrows: slight new bone formation within the implant (a score of 1); green arrowheads: fibrous connective tissue within the implant matrix; clear double arrows: cortical Haversian bone. **(B)** Animal D84-01 (SB). Left femoral condyle. Section T-1. Test article. Post-implantation day 83. Green circle: implant site outline; green arrows: mild new bone formation within the implant (a score of 2); the upper and middle areas of the implant are invested by periosteal and fibro-tendinous connective tissue (green arrowheads); clear double arrows: cortical Haversian bone. **(C)** Animal D84-01 (SB). Right femoral condyle. Section T-2. Test article. Post-implantation day 83. Green circle: implant site outline within the cancellous bone; green arrows: new bone formation throughout the implant (a score of 4); yellow arrowheads: bone in contact (a score of 1). **(D)** Animal D84-03 (SB). Right femoral condyle. Section T-1. Test article. Post-implantation day 83. Green circle: implant site outline within the cancellous bone; green arrows: new bone formation through most of the implant (a score of 4, confirmed by hematoxylin and eosin staining); green arrowheads: bone in contact (a score of 1). SB: Stevenel’s blue.

**FIGURE 5:**
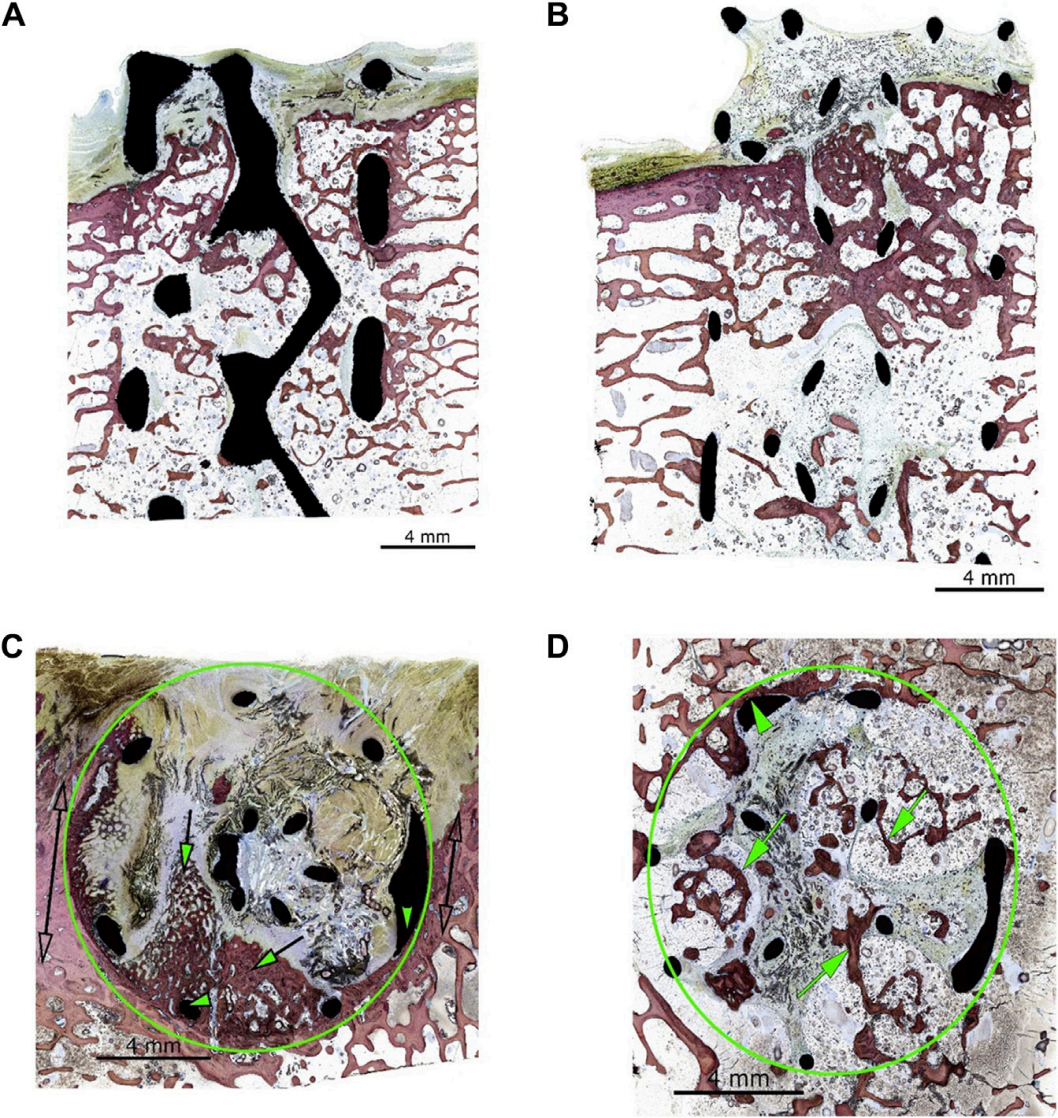
**(A)** Animal D84-03 (SB). Right femoral condyle. Section L-1. Test article. Post-implantation day 83. **(B)** Animal D84-04 (SB). Right femoral condyle. Section L-1. Control article. Post-implantation day 83. **(C)** Animal D84-02 (SB). Left femoral condyle. Section T-1. Control article. Post-implantation day 83. Green circle: implant site outline; green arrows: mild new bone formation within the implant (a score of 2); the upper portion of the implant is invested by periosteal and fibro-tendinous connective tissue; green arrowheads: bone in contact (a score of 2); clear double arrows: cortical Haversian bone. **(D)** Animal D84-02 (SB). Left femoral condyle. Section T-2. Control article. Post-implantation day 83. Green circle: implant site outline within the cancellous bone; green arrows: new bone formation throughout the implant (a score of 4). SB, Stevenel’s blue.

Using the grading system outlined in Tables 2, 3, the implants generally showed advanced osseointegration (scores of 2, 3, and 4 represented greater than 50% bone ingrowth, greater than 75% bone ingrowth, and diffuse bone ingrowth, respectively) and were characterized by the ingrowth of trabecular bone within the pores. New bone was undergoing woven-to-lamellar transition, which was consistent with ongoing ingrowth and maturation. On occasion, bone ingrowth was limited to the periphery or an angular fraction of the implant cage. This was generally observed in sections of tissue derived from the cortical edge of the implanted bone. New bone formation was accompanied by well-vascularized fibrous connective tissue throughout and by encroachment of fatty marrow peripherally. Histopathologic scores of bone directly in contact with the implant material (bone apposition) remained generally low in both groups (scores of 1 or 2) and showed no group-dependent trend. Osseointegration and bone implant apposition were comparable between the test and control groups; moreover, good hard tissue integration of the implant in this model, with widespread-to-extensive bone ingrowth, was typically observed. Bone maturation was ongoing at 12 weeks, Figures 4A–D, 5A show images of the histological results for the TA. Figures 5B–D show images of the histological results for the CA.

**TABLE 3:** Histological evaluation system for cell type/response associated with test/control articles.

Response	Score (phf = per high-powered [×400] field)				
	0	1	2	3	4
Polymorphonuclear cells	0	Rare, 1–5/phf	6–10/phf	Heavy infiltrate	Packed
Lymphocytes	0	Rare, 1–5/phf	6–10/phf	Heavy infiltrate	Packed
Plasma cells	0	Rare, 1–5/phf	6–10/phf	Heavy infiltrate	Packed
Macrophages	0	Rare, 1–5/phf	6–10/phf	Heavy infiltrate	Packed
Giant cells	0	Rare, 1–2/phf	3–5/phf	Heavy infiltrate	Packed
Necrosis/osteolysis	0	Minimal	Mild	Moderate	Marked
Infection	0	Minimal	Mild	Moderate	Marked
Fibrinous exudates	0	Minimal	Mild	Moderate	Marked
Neovascularisation	0	Minimal capillary proliferation focal, 1–3 buds	Groups of 4–7 capillaries with supporting fibroblastic structures	Broad band of capillaries with supporting structures	Extensive band of capillaries with supporting fibroblastic structures
Fibrocytes/fibroconnective tissue, fibrosis	0	Narrow band	Moderately thick band	Thick band	Extensive band
Fatty infiltrate	0	Minimal amount of fat associated with fibrosis	Several layers of fat and fibrosis	Elongated and broad accumulation of fat cells about the implant site	Extensive fat surrounding the implant

#### 3.2.3 Implant local biocompatibility

There was no-to-very minimal inflammatory response to the implant material in any of the animals or sites evaluated. Most of the implant surfaces were devoid of any inflammatory cell reaction, except for the presence of scattered macrophages and multinucleated giant cells. The implant material showed direct apposition with the fibrocellular tissue filling the implant spaces and occasional direct bone apposition (Figure 4C). In a few implants, there were small voids or fluid-filled spaces along portions of the implant (generally concave surfaces). These spaces were very narrow and may have resulted from soft tissue shrinkage upon fixation and/or processing. There was no necrosis or tissue degeneration along or around the implant material. The TA and CA were optimally tolerated in this model and were non-irritant.

#### 3.2.4 Drained lymph nodes

There were no abnormalities in any of the lymph nodes examined. None of the drained lymph nodes were reactive, and there was no evidence of inflammation or microparticulate derived from the procedure or implant material.

#### 3.2.5 Histomorphometry

**FIGURE 6:**
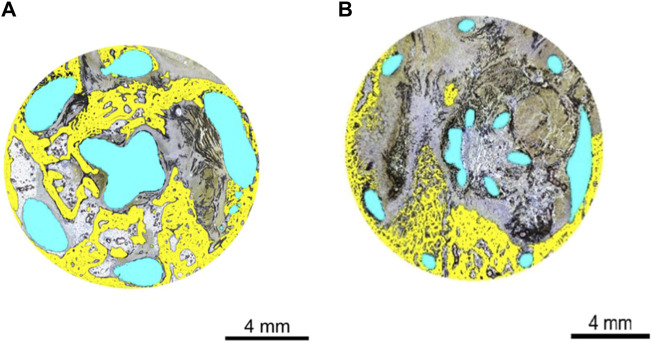
**(A)** Histomorphometry of animal D84-03 (SB). Right femoral condyle. Section T-1. Test article. Post-implantation day 83. Implant geometry is highlighted in blue. New osseous tissue formation is highlighted in yellow. **(B)** Histomorphometry of animal D84-02 (SB). Left femoral condyle. Section T-1. Control article. Post-implantation day 83. Implant geometry is highlighted in blue. New osseous tissue formation is highlighted in yellow. SB, Stevenel’s blue.

New bone area and bone apposition average values were very similar between the CA and TA (14.8% and 17.0%, respectively), reflecting widespread-to-extensive osseointegration. The implant area was approximately four times greater in the test group than in the control (23.0% and 6.3%, respectively), in keeping with the respective strut sizes. The soft tissue area was correspondingly slightly reduced in the test group (62.3%) compared with that in the control group (72.5%). The histomorphometry analysis can be seen in Figure 6.

#### 3.2.6 Micro-CT

**FIGURE 7:**
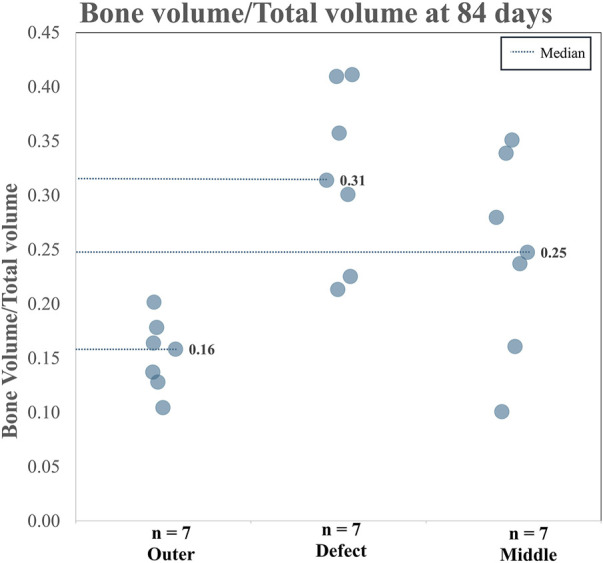
Combined BV/TV data for the TA and the CA across three analyzed regions.

**FIGURE 8:**
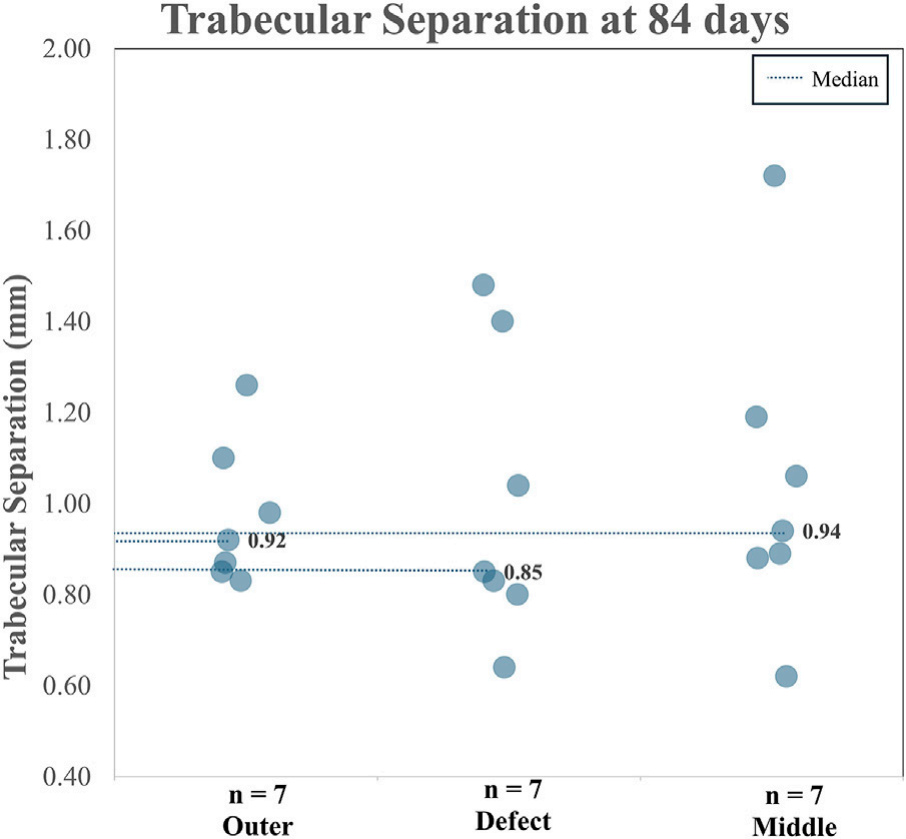
Combined Tb.Sp data for the TA and the CA.

**FIGURE 9:**
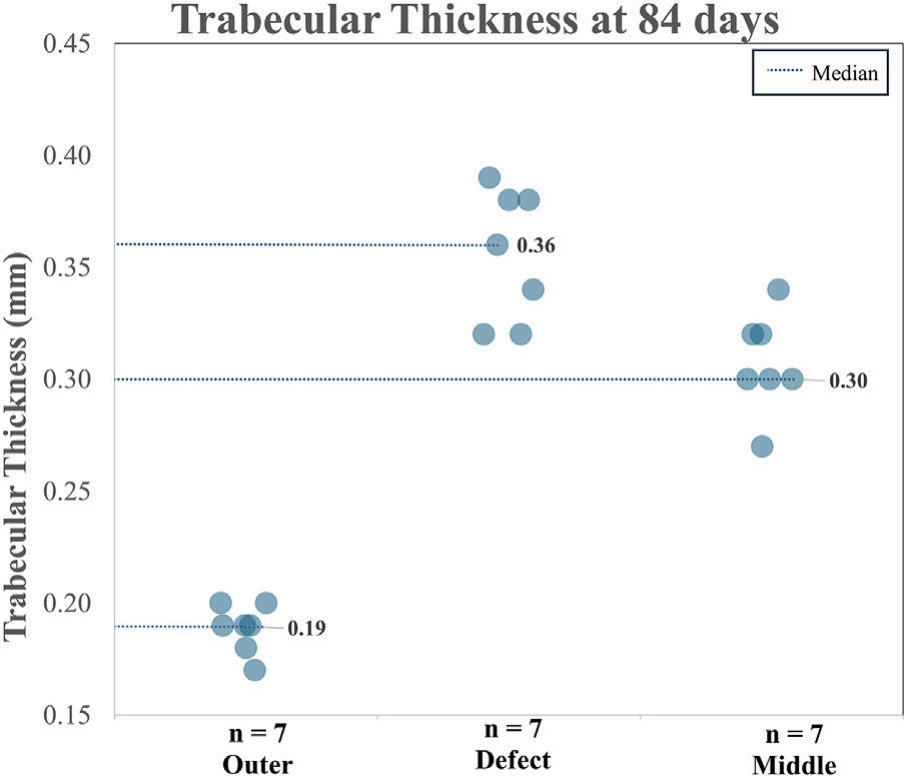
Combined Tb.Th data for the TA and CA across three analyzed regions.

When analyzing TA and CA (combined), for BV/TV and Tb.Th in the outer region *versus* the defect region, as well as the outer region *versus* the middle region, it is clear both the BV/TV and the Tb.Th were higher in the defect region than in the outer region. Additionally, BV/TV and Tb.Th were increased in the middle region than in the outer region. The BV/TV data across both the TA and CA in each region are shown in Figure 7. The BV/TV was higher in the defect/middle samples (test regions) than in the outer samples (control region), indicative of extensive bone growth into the porous architecture of the implant. Figure 8 shows the Tb.Sp in the outer, defect, and middle regions, respectively. No significant difference in Tb.Sp was observed between newly formed bone ingrowth and existing bone or between the test and control groups. Figure 9 shows Tb.Th across both the TA and CA in each region. We observed that Tb.Th was increased in the defect/middle (test) regions than in the outer (control) region. This was expected as new osseous tissue microarchitecture is predominantly composed of plate-like elements with inherently thicker trabeculae.

## 4 Discussion

Joint biomechanics and the anatomical location determine the specific mechanical requirements of an implant. AM allows for the inclusion of a lattice structure in the morphology of titanium-based implants. This architecture serves as a method for reducing stiffness to match that of local bone, with an elastic modulus in the range of 3.4–26.3 GPa [for Ti-6Al-4V scaffolds manufactured using LPBF (Weißmann et al., 2016)]. In aligning the mechanical properties of implants and tissue, the effect of stress shielding is minimized.

A further, key benefit of the customizable nature of 3D-printed cages is the ability to design an implant to conform to each patient’s anatomy. The literature suggests that the preservation of cortical integrity through anatomical design minimizes the risk of subsidence in weight-bearing joints due to increased surface area and the elimination of stress raisers (Burnard et al., 2019). Stable arthrodesis, with a solid fusion mass present throughout the structure of a titanium cage, has been widely reported in the literature for interbody fusion of the spine (Burnard et al., 2019). Additionally, Nwankwo et al. (2019) provided clinical results relating to a 5-year follow-up for a complex foot and ankle trauma case in which a patient was treated with a 3D-printed titanium cage (Nwankwo et al., 2019). The patient, a 46-year-old woman, opted for a limb salvage operation through arthrodesis of the tibia to the hindfoot using a titanium-based cage in conjunction with an intramedullary rod. Six months postoperatively, the patient could walk again without a need for ambulatory aids. At 60 months, CT scans indicated successful bone incorporation of the tibia, calcaneus, and talus into the cage, and the patient reported the restoration of 85% of her pre-injury functionality. Although a low American Orthopedic Foot and Ankle Society score (71 out of 100) was reported for this patient, this was attributed to a loss of hindfoot and sagittal motion (Nwankwo et al., 2019). These case studies provide a compelling argument for the integration of patient specific, AM, titanium-based implants into clinical practice.

Titanium-based cage implants have proven to be an effective measure for filling large bone cavities; however, the efficacy of these implants is contingent on extensive and widespread osseointegration (Distefano et al., 2023). Here, we evaluated the efficacy of the bilateral implantation of two Meshworks titanium-based, custom-made cages with two different cage strut thicknesses (TA/CA) and found that they were well-tolerated during the 12-week implantation study, with no adverse clinical signs or indication of pain/lameness being detected. At necropsy, there were no macroscopic observations that were directly attributable to the implanted cages. Microscopic evaluation demonstrated generally good integration of the implant, with widespread-to-extensive bone ingrowth, and good osseointegration and bone implant apposition. Bone maturation was ongoing at 12 weeks. Both the TA and CA were optimally tolerated in this model and were non-irritant. Additionally, there was no evidence of inflammation or microparticulate derived from the procedure or the implant material.

Histomorphometry results demonstrated that the soft tissue area in the test group was slightly reduced compared with that in the control group due to the larger strut thickness of the test group. A micro-CT analysis of the test and control groups combined showed that the BV/TV and the average Tb.Th were greater in both the defect and the middle region compared to surrounding adjacent tissue.

The results of this study suggested that additively manufactured titanium-based cages support osseointegration in the treatment of load-bearing defects that span cortico-cancellous regions. As such, LPBF titanium scaffolds represent promising osteoconductive biomaterials for both reconstructive surgery and traumatologic applications.

In summary, in this study, we found that titanium-based cage implants encouraged bone growth throughout the implant. No marked difference in bone formation was observed between the TA and CA, distinguished by variation in strut thickness. As expected, higher levels of bone ingrowth were observed in the endocortical region.

The animals were monitored throughout this 12-week study, and the implants were well-tolerated with no adverse clinical signs detected. Macroscopic examination of the tissue surrounding the area of implantation found no pathological indications that were inconsistent with the standard healing process following the mechanical trauma of a surgical procedure.

## Data Availability

The raw data supporting the conclusion of this article will be made available by the authors, without undue reservation.
